# Label-free detection of leukemic myeloblasts in hyaluronic acid

**DOI:** 10.1186/s13036-022-00308-6

**Published:** 2022-11-01

**Authors:** Suhyun Park, Hyueyun Kim, Minna Woo, Minsuk Kim

**Affiliations:** 1 Department of Pharmacology, College of Medicine, Ewha Womans University, Seoul, 07804 Republic of Korea; 2 Department of Medicine, Toronto General Hospital Research Institute and Division of Endocrinology and Metabolism, University Health Network, University of Toronto, Toronto, ON Canada

**Keywords:** Microfluidic system, Motion microscopy, Myeloid leukemia, Vibration

## Abstract

Chronic myeloid leukemia is generally required bone marrow biopsy for diagnosis. Although examining peripheral blood is less invasive, it has not been fully validated as a routine diagnostic test due to suboptimal sensitivity. To overcome this limitation, a number of methodologies based on microfluidics have been developed for sorting circulating tumor cells from peripheral blood of patients with leukemia.

In order to develop a more convenient method, we designed an analysis protocol using motion microscopy that amplifies cellular micro motions in a captured video by re-rendering pixels to generate extreme magnified visuals. Intriguingly, no fluctuations around leukemic myeloblasts were observed with a motion microscope at any wavelength of 0–10 Hz. However, use of 0.05% hyaluronic acid, one type of non-newtonian fluid, demonstrated fluctuations around leukemic myeloblasts under conditions of 25 μm/s and 0.5–1.5 Hz with a motion microscope.

Thus, the non-invasive detection of leukemic myeloblasts can offer a valuable supplementary diagnostic tool for assessment of drug efficacy for monitoring patients with chronic myeloid leukemia.

## Introduction

Leukemia is a type of blood cancer which generates the malignant white blood cells in the human body [1]. These abnormal cells can ultimately lead to failure of the immune system and can compromise other haematopoietic lineages causing low red blood cells and platelets [2]. Moreover, the malignant cells can proliferate in immune organs such as the spleen and lymph nodes causing their massive enlargement or can invade into other non-immune tissues such as the liver and the kidney which can often be fatal [3]. Therefore, early detection and intervention in leukemia management is an important factor for successful treatment [4]. The gold standard for diagnosis of leukemia is bone marrow biopsy [5]. However, less invasive and efficient screening by cytogenetics, fluorescence in situ hybridization, microfluidic-based assay has been applied in peripheral blood cells for leukemia detection [4, 5]. To develop a more convenient method, we have developed and reported a novel method to visualize specific micro-vibration of tumor cells in continuous flow [6].

Motion microscopy is a digital software that quantifies micro motions from videos by generating new pictures whereby the motions are amplified sufficiently for detection [7–10]. The principle is to magnify micro signals of motion that can be stored in the pixel of a digital camera. Therefore, more pixels covering the object would generate better signals for extraction [10]. For every pixel at location (x, y), time t, scale r, and orientation θ, spatial local phase information was combined in different sub-band of frames using the least squares objective function [6, 7, 9, 10], $\mathrm{argmin}{\sum }_{i}{A}_{ri,\theta i}^{2}\left[\left(\frac{{\partial \phi }_{ri,\theta i}}{\partial x},\frac{{\partial \phi }_{ri,\theta i}}{\partial y}\right)\left(u,v\right)-{\Delta \phi }_{ri,\theta i}\right]$. In a previous study, we amplified the movement of breast tumor cells using motion microscopy and this was referred as cellular trail [6]. The principle behind this phenomenon is the composition of proteins on the surface of tumor cells which induces distinct fluid friction [6]. Leukemic myeloblast surface may also raise the fluid resistance and is investigated in this study.

We therefore hypothesized that motion microscopy can be used to detect leukemic myeloblasts. To this end, we analyzed wavelength and flow rate profiles in leukemic myeloblasts using motion microscopy.

## Materials and methods

### Cell lines and culture

Human leukemia cell line K562 was cultured in RPMI 1640 (A2494201, Gibco, USA) supplemented with 10% heat-inactivated fetal calf serum (16,000,044, Gibco, USA), 2 mM glutamine, 20 mM Hepes (pH 7.5) and maintained at 37 ℃ under an atmosphere of 95% O_2_ and 5% CO_2_. To prepare leukocyte, human whole blood (HUMANWBK2, BIOIVT, USA) was mixed with a separation medium (C-44010, Sigma-Aldrich, USA) and centrifuged at 400 × g for 15 min. Peripheral blood mononuclear cells (PBMCs) were obtained from individuals with chronic myeloid leukemia (PBMNC005C-CML PBMC, BIOIVT, USA) using Institutional Review Board (IBR) approved consent forms and protocols.

### Microfluidic device and motion microscopy

Microfluidic devices (Polydimethylsiloxane chip, Microfit, South Korea) were placed on the stage of an inverted microscope and the fluid flow was controlled by individual syringe pumps (BS-9000–12, Braintree scientific, USA). The microfluidic device and syringe pumps were connected by polythene tubing (PE10, Braintree scientific, USA) with an inner diameter of 0.28 mm. Prior to each experiment, isopropanol (W292907, Sigma-Aldrich, USA) was flushed through the whole microfluidic device to remove air bubbles in the channel followed by 1 X PBS (10,010,023, GIbco, USA) wash for 30 min. Leukemia cells or leukocytes were then introduced to the device at a flow rate of 10–30 μm/s and video files were recorded through the inverted microscope at 1200 × 512 pixels and 500 frames per second. The recorded videos were uploaded to lambda vue (https://lambda.qrilab.com/site/) and the magnification type was selected in colour mode, with amplification ratio of 20, and wavelength was selected from 0.1 Hz to 10 Hz in conversion condition.

### Quantification of cellular trail intensity

The obtained images were converted to 8-bit format in order to perform uncalibrated optical density. After conversion, the background was subtracted through the rolling ball radius method and cellular trails were individually selected. The area of histograms were obtained and quantified by ImageJ (Java-based image-processing and analysis software). Data were acquired as arbitrary area values.

### Optical tomographic microscope

Green light (λ = 520 nm, exposure 0.2 mw/mm^2^) from a laser diode was splitted into cells and reference beam at Nanolive (3D cell explorer, Switzerland). Cells were illuminated with a laser beam inclined at 45° which rotates around the sample 360°. Holographic images were recorded on a digital camera by combining the beam that had passed through the cells with the reference beam. The 3D cell images were recorded up to 30 μm depth of reconstruction.

### Viscosity measurement

Hyaluronic acids (75,043, Sigma-Aldrich, USA) and 1 × PBS (10,010,023, GIbco, USA) were slowly mixed with a blender until completely liquefied. Viscosity for 0.01, 0.02, or 0.05% hyaluronic acids was measured with a cone-and-plate digital viscometer (ASTM D4287, Industrial Physics Inks & Coatings, Netherlands). Shear rates were generated by rotating the brush around 750 rpm and non-newtonian fluid properties were determined.

### Cell viability

K562 and leukocytes were treated with 0.01, 0.02, 0.05, or 0.1% of hyaluronic acid for 12 h at 37 ℃ under an atmosphere of 95% O_2_ and 5% CO_2_. Using CCK-8kit (ab228554, Abcam, USA), tetrazolium was converted to formazan by dehydrogenase activity from mitochondria of living cells, and cell viability was determined following detection of optical density at 460 nm.

### Western blot

Briefly, human leukemic myeloblasts (K562) were homogenized in ice-cold lysis buffer. After centrifugation at 5,000 g for 20 min, protein content of the supernatant was quantified using a Bradford protein assay. Samples were diluted, boiled with sample loading dye, and 100 mg were loaded in SDS-PAGE (4561033EDU, Bio-Rad). After blotting, membranes were blocked in 5% skim milk (70,166, Sigma-Aldrich) in PBS containing 0.1% Tween-20 (P1379, Sigma-Aldrich). Membranes were incubated with antisera directed against *CDC42* (1:1000; 2462, Cell Signaling, USA), and then with secondary antibodies (mouse-specific HRP-conjugated antibody or rabbit-specific HRP-conjugated antibody). Bands were visualized using ECL detection kit (32,106, Thermo Scientific) and quantified by densitometry. Blots were stripped and re-exposed to detect *TUBB* (1:1000; 2125, Cell signaling, USA) as housekeeping protein.

### Fluorescence microscope

Briefly, PBMCs were placed in 10% formalin for 3 h and incubated with antisera against FITC-conjugated CD117 (1:400; ab119107, abcam, USA). After washing with PBS, cells were visualized using Zeiss LSM 510 confocal microscope (Carl Zeiss, German).

### Statistical analysis

Values are means ± SE. The significance of differences was determined by a two-way analysis of variance (ANOVA), or a one-way ANOVA followed by a Bonferroni post-hoc analysis where appropriate. Differences were considered significant when *P* < 0.05.

## Results and discussion

### Design to visualize oscillating movement of leukemic myeloblasts

**Fig. 1:**
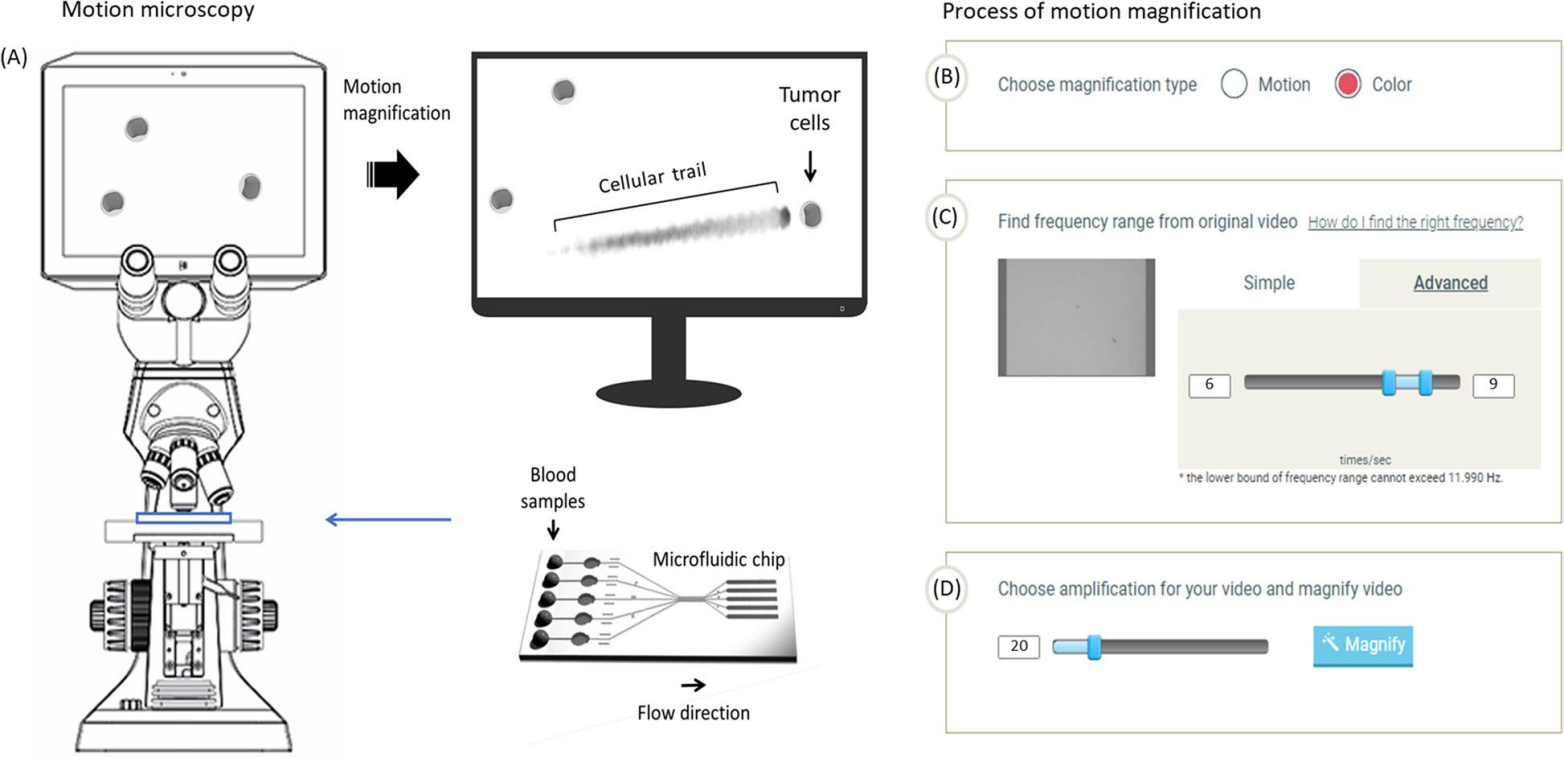
Contact free system to detect leukemic myeloblasts using cellular motions. (**A**) Schemata of the experimental setup of microfluidic device and motion microscope. Human leukemic myeloblasts were subjected to the micro fluidic device at a flow rate of 25 μm/s and video recording files were obtained from the microscope at 1200 × 512 pixels at 500 frames per second. (**B**) The obtained videos were entered at lambda vue and color modes were selected in magnification type. After setting the wavelength between 0.1 ~ 10 Hz (**C**), cellular motions were amplified 20 times (**D**) and magnified images obtained

To overcome the difficulty of focusing on continuous recordings of multiple moving cells, we had cells rolling on the surface using microfluidics. Human leukocytes and leukemic myeloblasts were subjected to flow on a polydimethylsiloxane based microfluidic channel at a flow rate of 25 μm/s and then recorded at 1200 × 512 pixels at 500 frames per second (Fig. 1A). Micro movements of cells were amplified by a motion microscope and detailed settings were in color mode and magnification type (Fig. 1B), 0.1 to 10 Hz in wavelength (Fig. 1C), and 20 times in amplification rate (Fig. 1D). The modified images were obtained through the process listed above (Fig. 1B-D).

### Non-newtonian fluid elevates the sensitivity of micro-vibration

**Fig. 2:**
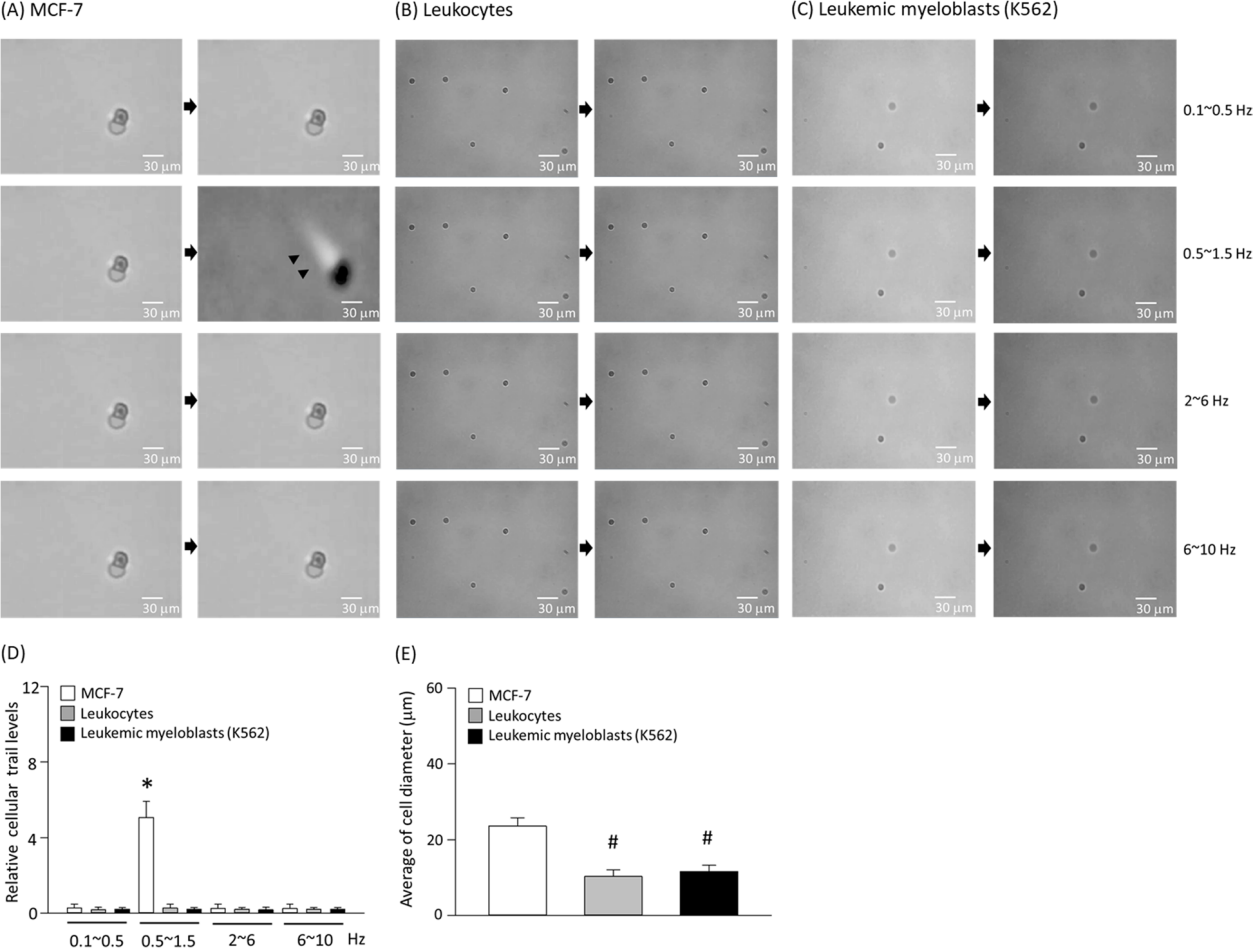
Cellular trails were not observed in K562 cells. (**A**) MCF-7, (**B**) leukocytes, or (**C**) K562 cells were subjected to the microfluidic device at a flow rate of 25 μm/s and the images were converted by a motion microscope at 0.1–10 Hz. Cellular trails were indicated by arrowheads. (**D**) Intensity levels of cellular trails in motion magnified videos between 0.5–1.5 Hz. (**E**) Diameters of MCF-7, leukocytes, or K562 cells. Results are the means ± SE of 6 experiments in each group. *Significantly different from motion magnified videos at 0.1 Hz–0.5 Hz, *P* < 0.05. ^#^Significantly different from MCF-7 cells, *P* < 0.05

**Fig. 3:**
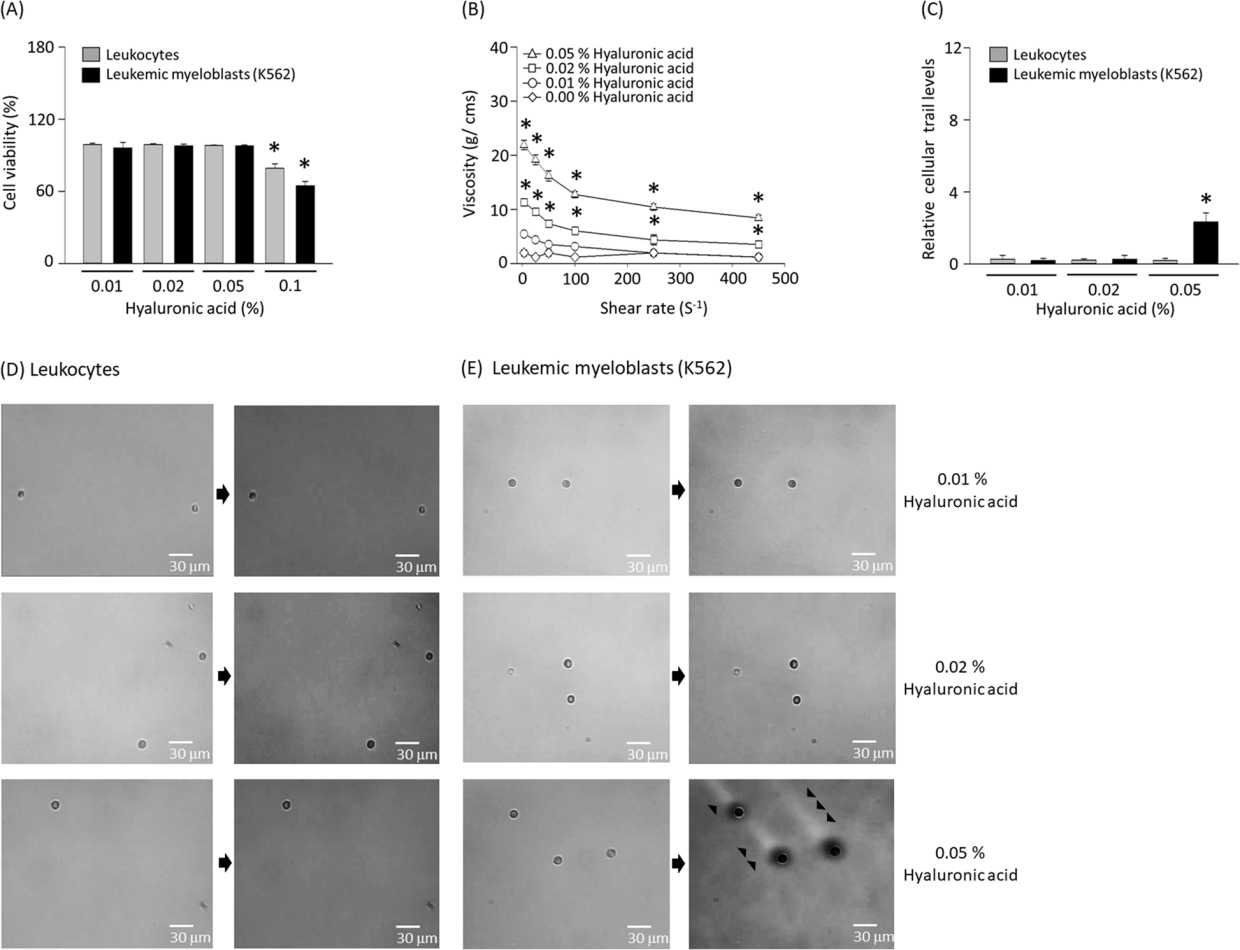
Hyaluronic acid makes cellular trails of K562 clearly visible. (**A**) K562 and leukocytes were treated with 0.01, 0.02, 0.05, or 0.1% of hyaluronic acid for 12 h and cell viability was measured with CCK-8 kit. (**B**) Viscosity of hyaluronic acid was measured with a cone-and-plate digital viscometer at six different shear rates. The viscosity values for 0.01, 0.02, 0.05% hyaluronic acids were followed a pattern of a non-newtonian fluid. (**C**) The intensity levels of cellular trail were determined at various hyaluronic acid concentrations. The images of micro motions (arrow heads) of (**D**) leukocytes, or (**E**) K562 cells were converted by a motion microscope at 0.5–1.5 Hz. Results are the means ± SE of 6 experiments in each group. *Significantly different from 0.01% hyaluronic acid treated group, *P* < 0.05

Previously, a motion microscope detected vibrations of breast cancer cells (Fig. 2A) and named it as cellular trail [6]. MCF-7 cells were clearly distinguishable from leukocytes using the motion microscope under condition of 0.5 to 1.5 Hz (Fig. 2B). However, cellular trails were not observed in K562 cells which are similar in size to leukocyte at various wavelengths of 0.1 to 10 Hz (Fig. 2C-E). Vibrational flow has been known to be amplified in non-newtonian fluids [11]. We therefore hypothesized that non-newtonian fluids can be used to detect tiny tumor cells in motion microscope. As a biomaterial, hyaluronic acid was used among the non-newtonian fluids [12]. Cell viability of leukocyte and K562 was measured while increasing the concentration of hyaluronic acid, and cytotoxicity was minimal at concentrations below 0.1% hyaluronic acid (Fig. 3A). Viscosity of a fluid is a measure of its resistance to gradual deformation by force or tension [13]. Therefore, unlike newtonian fluids, non-newtonian fluids have a change in viscosity following shear stress [13]. By measuring the viscosity of hyaluronic acid according to shear stress, the characteristic of non-newtonian fluid was confirmed at a concentration of 0.01 to 0.05% (Fig. 3B). Using hyaluronic acid with a concentration of 0.01 to 0.05%, the presence of cellular trails was observed using a motion microscope at 0.5 to 1.5 Hz (Fig. 3C). Leukocyte did not show cellular trails even with hyaluronic acid as a fluid (Fig. 3D). Intriguingly, a distinct cellular trail of K562 was observed at a concentration of 0.05% hyaluronic acid (Fig. 3E).

### Cellular trail of K562 under various conditions

**Fig. 4:**
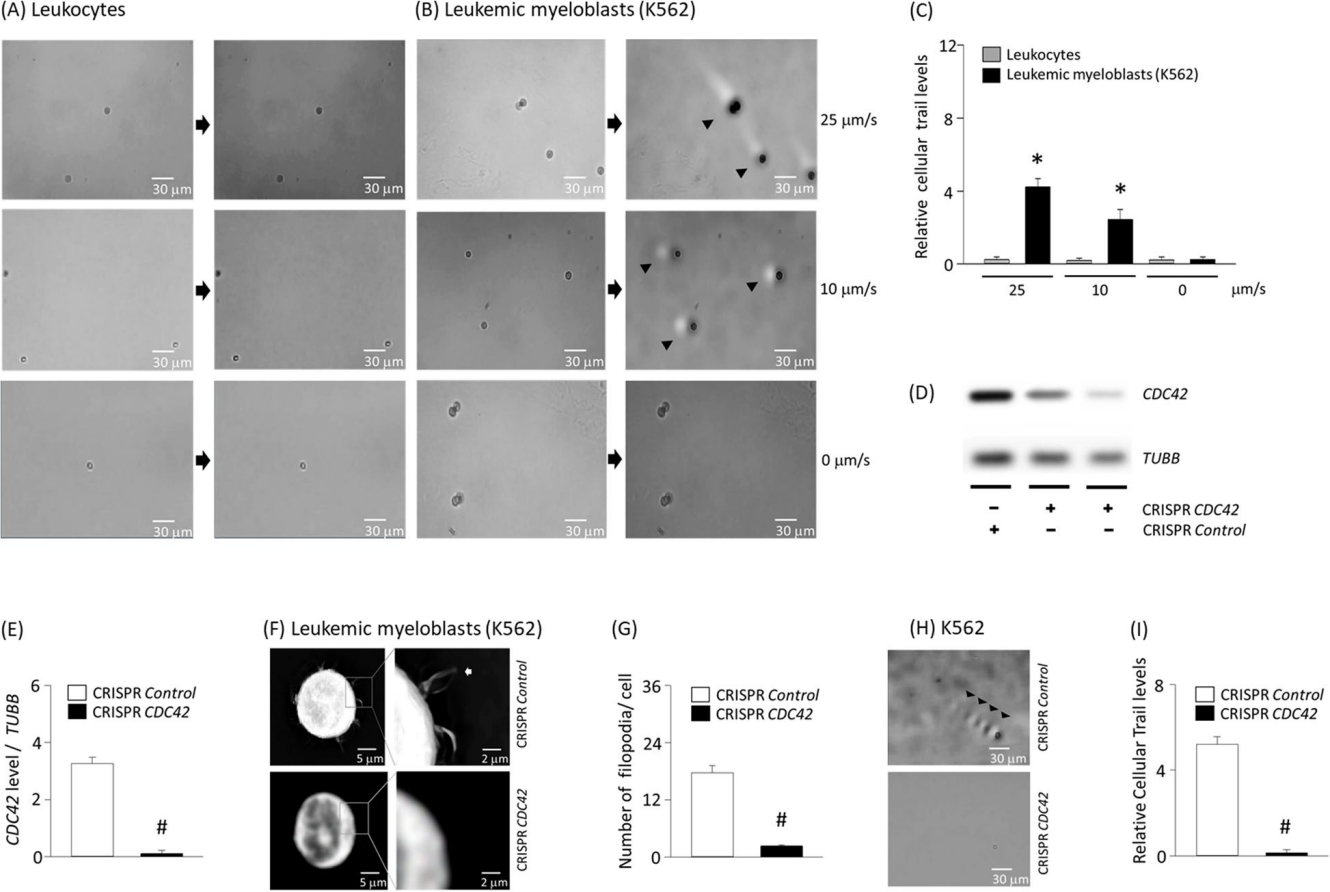
Role of flow rate and filopodia on the cellular trail. (**A**) Leukocytes or (**B**) K562 cells were subjected to the microfluidic device at various flow rates of 25 μm/s, 10 μm/s, or 0 μm/s and cellular trails (arrow heads) were determined. (**C**) The intensity levels of cellular trails were determined in motion magnified videos. Tumor cells were transfected with CRISPR-*CDC42* or CRISPR-*control*. (**D**) and (**E**) Proteins were extracted from each of the cell lines and expression of *CDC42*, or *TUBB* were determined using western blotting. (**F**) Holographic images were recorded by combining laser (λ = 520 nm) that had passed through the cells with up to 30 μm depth of reconstruction. From the 3D images, the outer surfaces of cells were described using optical tomographic microscope. (**G**) The number and distribution of filopodia on K562 cells. (**H-I**) Transfected cells were subjected to the microfluidic device and movements were magnified and cellular trails were assessed. Results are the means ± SE of 6 experiments in each group. *Significantly different from cellular trail of leukocytes, *P* < 0.05. ^#^Significantly different from CRISPR-*control* transfected cells, *P* < 0.05

We next examined other potential parameters that may affect changes in cellular trails in motion microscopy. One such factor is a frictional force of the cell surface following flow rates [6]. Therefore, to assess its effect, changes in flow rates were given at an amplification wavelength from 0.5 to 1.5 Hz in 0.05% hyaluronic acid (Fig. 4A and B). In an environment with a decelerated flow rate, 10 μm/s, the intensity of cellular trails was reduced two-fold (Fig. 4C). Moreover, when the flow velocity was reduced to zero, cellular trails of the K562 cells disappeared. Unlike tumor cells, cellular trails were not detectable in leukocytes at a flow rate of 0–25 μm/s. This allows tumor cells to be clearly distinguished from leukocytes in the blood of patients with leukemic myeloblasts under the same conditions of motion microscopy. Moreover, we hypothesized that filopodia on the surface of K562 cells can affect the intensity of cellular trails. *CDC42* has been known to be an important protein in the production of filopodia [14]. To elucidate the relationship between filopodia and cellular trail, *CDC42* level were lowered using transfection of CRISPR-Cas9 plasmid (Fig. 4D and E). Remarkably, deletion of *CDC42* significantly reduced the number of filopodia and intensity of cellular trails (Fig. 4F-I).

### Motion microscope increases sensitivity of detecting leukemic myeloblasts using non-newtonian fluid

**Fig. 5:**
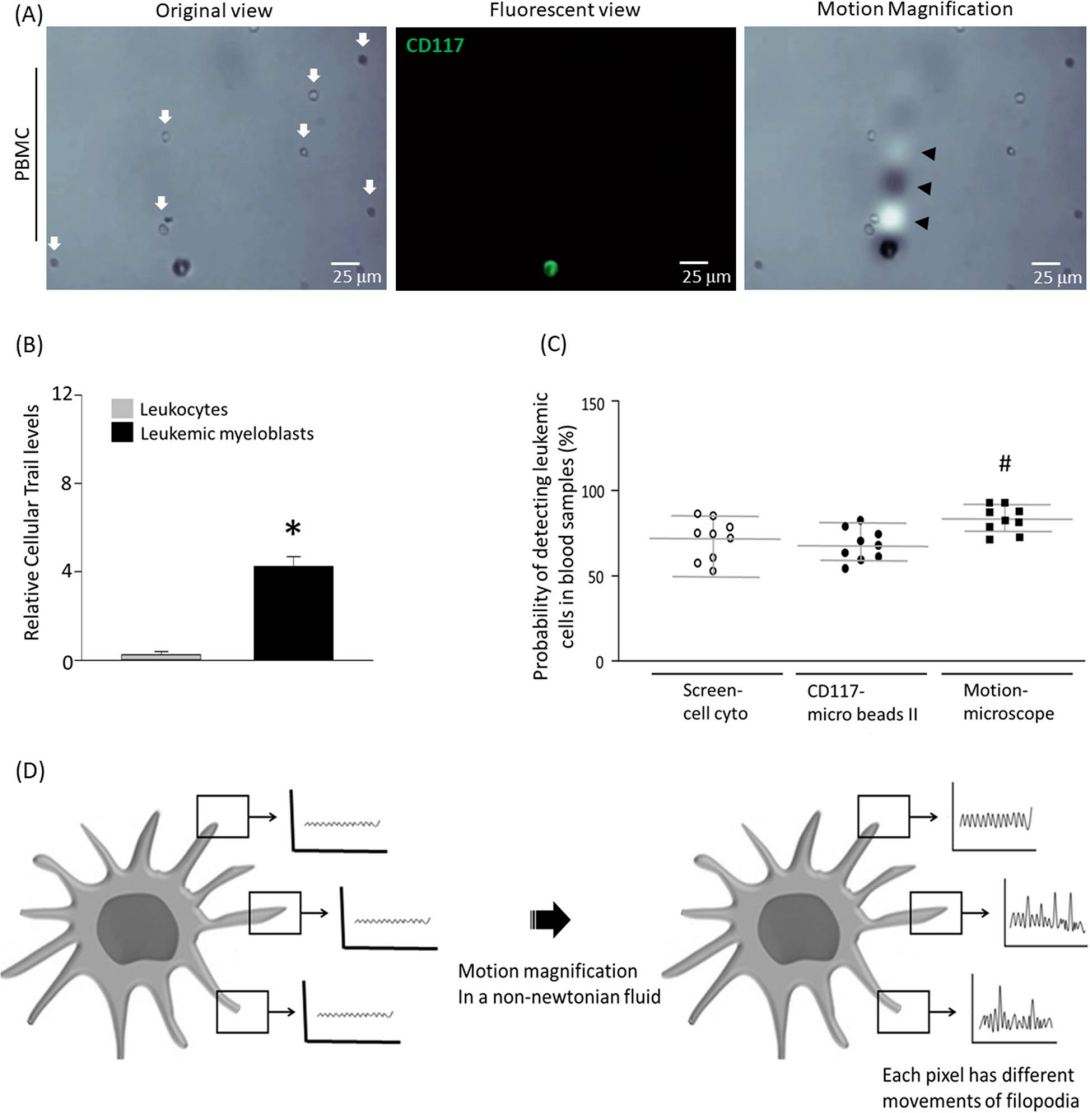
Potential use of motion microscopy for myeloid leukemia detection. (**A**) Detection of leukemic myeloblasts by cellular trails in PBMCs obtained from patients with myeloid leukemia. Leukemic myeloblasts were immunostained with CD117 antibody using fluorescence microscopy. Leukocytes are indicated by arrows. (**B**) The intensity level of cellular trails was determined in motion magnified videos. (**C**) Comparison with the size-based filtration system (screen cell cyto), immunoprecipitation using CD117 antibody, or motion microscope to detect leukemic myeloblasts in human blood samples. (**D**) Using a non-newtonian fluid, motion microscope is able to detect oscillating movement of leukemic myeloblasts through filopodia under condition of 25 μm/s and 0.5–1.5 Hz. Results are the means ± SE of 6 experiments in each group. *Significantly different from cellular trail of leukocytes, *P* < 0.05. ^#^Significantly different from screen cell cyto method, *P* < 0.05

We next assessed peripheral blood mononuclear cells (PBMC) of patients with leukemia under conditions of 25 μm/s and 0.5–1.5 Hz in 0.05% hyaluronic acid (Fig. 5A). Tumor cells were stained with CD117 antibody and confirmed using fluorescence microscopy. Table 1 illustrates PBMC information of leukemia patients. We found that cellular trails can be clearly distinguished between tumor cells and leukocytes (Fig. 5B). Method of immunoprecipitation or cell size difference was compared to that of motion microscopy (Fig. 5C). Total numbers of leukemic myeloblasts were counted manually using fluorescence microscopy. The immunoprecipitation or conventional size differential detection method yielded a sensitivity of 68%–87%, whereas motion microscopy method detected tumor cells with a sensitivity of 92%-97%. Moreover, the motion microscopy method yielded not only a higher detection rate but was also more consistent, while the immunoprecipitation or size-differential method had a large variation in results (Fig. 5C). Overall, filopodia of leukemic myeloblasts appeared to be essential in affecting the cellular trails and tumor cells were clearly distinguishable from leukocytes using the motion microscope under condition of 25 μm/s and 0.5–1.5 Hz in 0.05% hyaluronic acid (Fig. 5D).

## Conclusion

The purpose of the current experiment is to develop rapid diagnosis of chronic myeloid leukemia through visualization of microscopic vibrations. As leukemic myeloblasts have similar physical characteristics compared to leukocytes, it was difficult to detect using micro-vibration. However, we were able to overcome this limitation with the application of hyaluronic acid, which is a non-newtonian fluid. Moreover, motion microscope can detect leukemic myeloblasts more rapidly with higher sensitivity than conventional methods. Together, we offer a novel tool for detection of chronic myeloid leukemia which may be used for assessment of drug efficacy and physical characteristics of leukemic myeloblasts for further research.

**Table 1:**
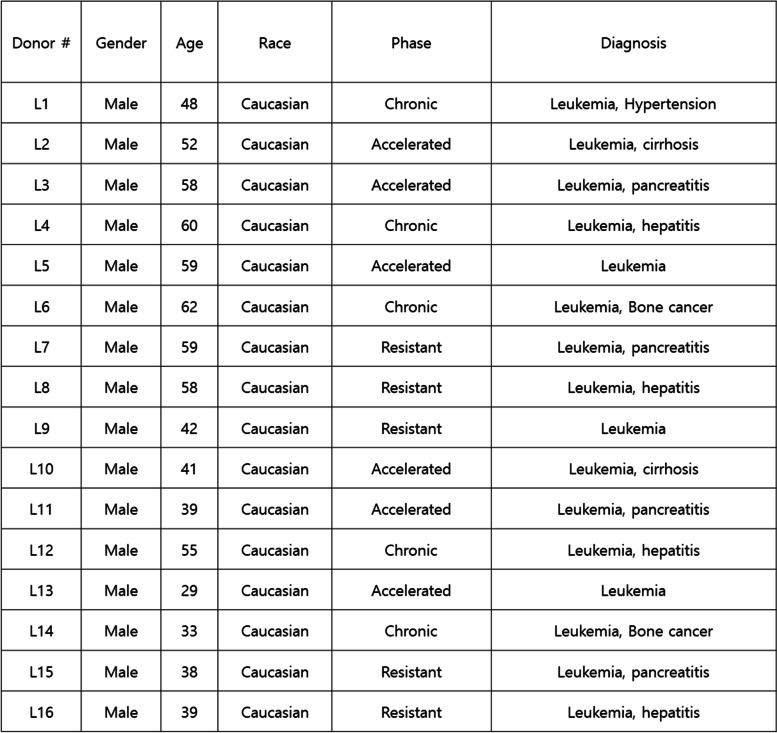
Detailed patient information

## Data Availability

All data supporting the findings of this study are available within the paper.
